# The Neural Correlates of the Abnormal Implicit Self-Esteem in Major Depressive Disorder: An Event-Related Potential Study

**DOI:** 10.3389/fpsyt.2022.822677

**Published:** 2022-07-04

**Authors:** Chen-guang Jiang, Heng Lu, Jia-zhao Zhang, Xue-zheng Gao, Jun Wang, Zhen-he Zhou

**Affiliations:** ^1^Department of Psychiatry, The Affiliated Wuxi Mental Health Center of Nanjing Medical University, Wuxi, China; ^2^3 Grade 2019 Class 6, Basic Medicine College of Jinzhou Medical University, Jinzhou, China

**Keywords:** major depressive disorder, implicit self-esteem, event-related potentials, go/no-go association task, neural mechanism

## Abstract

Implicit self-esteem (ISE) has been considered a critical factor in the development and maintenance of major depressive disorder (MDD). Further investigating the event-related potential (ERP) characteristics underlying abnormal ISE in MDD would be helpful for understanding the neural mechanism of MDD. For this purpose, 32 MDD patients and 31 age- and sex-matched healthy controls (HCs) were enrolled in this study. The Rosenberg Self-Esteem Scale (RSES) was used to evaluate explicit self-esteem (ESE), and a self-esteem go/no-go association task (GNAT) was used to assess ISE. Electroencephalograms were synchronously recorded when performing the self-esteem GNAT. Behavioral data and ERP characteristics under different conditions were analyzed and compared within and across groups. The results showed that compared to HCs, MDD patients had significantly lower RSES scores and *self*-D scores of GNAT, which reflected lower levels of ESE and ISE, respectively. No significant correlation was found between RESE and *self*-D scores, and only RESE scores were significantly negatively correlated with the Hamilton Depression Rating Scale (HAMD) score. The averaged centroparietal go-P3 amplitude under the *self-positive* condition was significantly smaller in MDD than in HCs. Moreover, HCs had a significantly larger average centroparietal go-P3 amplitude in *self-positive* than in *self-negative* conditions, while this pattern was opposite in the MDD group. The neural activity patterns for *other* conditions were similar between MDD and HCs. Our results suggested that patients with MDD have a decreased level of both ESE and ISE, and ISE might be more independent of clinical symptoms. Decreased neural processing that implicitly associate *self* with *positive* conditions (and relatively increased implicit association between *self* and *negative* conditions) might be important neural correlates for abnormal ISE in MDD.

## Introduction

Major depressive disorder (MDD) is one of the most common and disabling mental disorders worldwide. The link between MDD and negative clinical outcomes such as suicidality is strong, and people at high risk for suicidal behavior usually approached suicide through many ways (1). Proper self-esteem, that is, an appropriate and positive subjective evaluation of one’s own worth, is of great importance in maintaining a person’s mental health and wellbeing. Nevertheless, people with MDD often have low self-esteem, which is typically characterized by self-exclusion, self-denial, and self-contempt (2).

Self-esteem can be divided into two different psychological constructs, namely, explicit self-esteem (ESE) and implicit self-esteem (ISE). The former can be assessed using simple measurement strategies like self-reported questionnaires [e.g., Rosenberg Self-Esteem Scale (RSES)] (2). ISE, however, is thought to be outside of conscious control and cannot be realized through introspection. Greenwald and Banaji first proposed the definition of ISE, which grew out of the dual signal system (3). Distinct from ESE, ISE refers to a person’s disposition to evaluate themselves positively or negatively in a spontaneous, automatic, or unconscious manner. Accordingly, ISE is expected to unveil aspects of *self* that are not captured by ESE.

Both ESE and ISE have a relationship with MDD. It has been repeatedly reported that people with MDD often have lower ESE than the general population, while studies regarding ISE in MDD have yielded inconsistent results (4–9). It is worth noting that some evidence indicated that ISE may be more important than ESE as a target for interventions to prevent the recurrence of MDD. For instance, a previous study reported that ISE, but not ESE, could predict future depressive symptomatology (10). Another study also found that ISE is an important variable of vulnerability for MDD relapse (11). Recently, a 3-year follow-up study with a large sample size further confirmed that ISE could predict recurrence of depression even when statistically controlling for confounding factors at baseline, while the prediction value of ESE is relatively smaller (4). Interpretation of abnormal ISE was varied in previous studies. Some stated that lower ISE in MDD would be the marker of how deeply negative self-views are internalized (7), but some hold that increased positive implicit associations with others would have also played an important role in MDD (12). Taken together, further studies on characteristics of ISE in patients with MDD are warranted. In the present study, we aimed to verify the characteristics of ISE in clinically diagnosed MDD patients. Given that ISE is thought to be outside of conscious control and cannot be realized through introspection, it may represent more stable and deep-seated part of self-esteem than ESE. Hence, we expected that ISE would be less correlated with depressive symptom severity than ESE.

On the other hand, the neural mechanism behind abnormal ISE in MDD is far from being clarified. Event-related potential (ERP) is a powerful technique in neuroscience that can simultaneously provide information on neural electrical activity during behavioral tasks. Due to its high temporal resolution up to milliseconds, ERP may have advantages even over functional magnetic resonance imaging (fMRI) for studying ISE because ISE is detected using experimental paradigms requiring rapid response, such as the Implicit Association Test (IAT). In fact, the ERP technique has been adopted by many researchers to study the neural correlates of ISE. For instance, by combining the ERP and IAT, a study (13) reported that participants demonstrated a more positive ERP deflection between 350 and 450 ms after the onset of *self*-stimuli in congruent conditions (*self-positive*) than in incongruent conditions (*self-negative*). Another study combined ERP with the go/no-go association task (GNAT), a variant of IAT, and found that positive ISE was manifested on neural activity around 270 ms after the presentation of self-relevant stimuli (14). Notably, these studies were carried out in the general population, rather than MDD patients. Meanwhile, studies have found differences between healthy controls (HCs) and subclinical depressive individuals. As a recent study reported (15), relative to people without depressive symptoms, the neural activity pattern during the self-esteem IAT was reversed in dysphoric participants. This left an open question of whether clinically diagnosed patients with MDD have similar neural activity patterns.

It is considered that self-referential processing has a close relationship with self-esteem (16), and several studies have explored the implicit self-referential processing in patients with MDD. For instance, Dainer-Best et al. found that sustained attention involvement was related to the increased negative self-referential processing in patients with MDD (17). Recently, Benau et al. found that depressive participants were more likely to endorse negative self-referent sentences, and this could be reflected as larger late positive potential (LPP) to negative stimuli (18). It should be noted that the self-referent encoding task (SRET) or adapted SRET used in these studies is distinct from the IAT or the GNAT. SRET is an affective decision task in which participants make binary-choice decisions on whether positive and negative words or sentences are self-descriptive (19). In other words, SRET, at least in part, requires participants to consciously, rather than implicitly, judge whether the stimulus is relevant to them. Therefore, it remains unclear whether the findings obtained using the SRET paradigm well reflect the ISE characteristics of people with MDD.

In the present study, we aim to reveal the potential neural correlates of abnormal ISE in clinically diagnosed MDD patients by combining a self-esteem GNAT paradigm and the ERP technique. According to Beck’s cognitive theory (19), uncontrollable automatic biases toward negative information play an important role in the development and maintenance of depression. Therefore, MDD patients are likely to have more spontaneous attention toward a negative stimulus and stronger *self-negativity* association. In addition, anhedonia, an inability to experience pleasure, is common among MDD patients (20). From this perspective, positive stimuli may capture less attention than negative stimuli and thus induce less neural activity in this population. Based on the aforementioned theory and findings, we hypothesized that when performing the GNAT, MDD patients would have much smaller amplitudes of ERP components that reflect attention and emotional processing (like P3/LPP) than their counterparts; moreover, MDD patients *per se* would have lower ERP amplitudes to *self* items under *positive* conditions than under *negative* conditions, which represents a neural process bias to implicit association between *self* and *negativity*.

## Materials and Methods

### Time and Setting

The present study was carried out from 1 July 2018 to 31 March 2021, in the Affiliated Wuxi Mental Health Center of Nanjing Medical University, Wuxi city, the People Republic of China. This study was approved by the Ethics Committee of the Wuxi Mental Health Center and conducted in accordance with the Declaration of Helsinki.

### Participants

All MDD patients were recruited from inpatients of the Wuxi Mental Health Center. The inclusion criteria were (a) individuals meeting the criteria of MDD according to the Diagnostic and Statistical Manual of Mental Disorders, Fourth Edition (DSM-IV) (21), (b) Chinese Han aged 18–65 years, and (c) volunteer to participate in this study. The exclusion criteria were individuals (a) meeting the criteria of any other mental disorder according to DSM-IV, (b) received electroconvulsive therapy in the last 24 weeks, (c) having neurological illness or other severe physical illness as determined by clinical evaluations and medical records, (d) having nicotine/other substance misuse or dependence, and (e) having taken any medication known to affect cognition within the past 2 weeks. All HCs were recruited from the local residential communities through advertisement. The inclusion criteria of HCs were (a) individuals meeting no criteria of any kind of mental disorder according to DSM-IV, and (b) and (c) criteria same as the MDD group. The exclusion criteria of HCs were the same as (c) to (e) criteria as the MDD group.

Both MDD patients and HCs provided written informed consent to participate in this study. For MDD patients whose capacity to consent was compromised, we obtained consent from their next of kin or guardians. Each participant was compensated 300.00 Chinese yuan (CNY).

### Clinical Assessments

The Hamilton Depression Rating Scale (HAMD, 24-item version) (22) was employed to evaluate the severity of depressive symptoms of MDD patients by an experienced senior psychiatrist. Higher HAMD scores indicate more severe depression. The RSES (2), a widely used ESE evaluation tool, was employed for all participants. This self-rating scale has 10 items regarding the overall feelings of self-worth and self-acceptance. The RSES score ranges from 4 to 40, with higher scores suggesting a higher level of ESE.

### Self-Esteem Go/No-Go Association Task

We employed a self-esteem GNAT paradigm to study the characteristics of ISE. Relative to the IAT, the GNAT may have some unique advantages. For instance, the GNAT relies on fewer blocks, which can reduce cognitive confounds like task switching, which frequently occurs in the IAT (23). Moreover, the GNAT requires the participants to press only one button (only one category and one evaluative attribute) over two buttons, which can not only detect the approach function but also the inhibition ability. In addition, the GNAT is more flexible in measuring automatic cognition (24). Despite studies comparing the IAT and GNAT have confirmed that they can effectively measure the automatic self-evaluation and are both valid measurement tools for ISE (23), a recent study has shown that the GNAT may detect some implicit bias that are not easily detected by the IAT (24).

Referring to a recent study in this field (13), we programmed a self-esteem GNAT paradigm by E-Prime 3.0 software (Psychology Software Tools Incorporated, Pittsburgh, United States) (25) to assess the level of ISE. Stimuli included 90 Chinese words: 40 *positive* words, 40 *negative* words, 5 words served as the self category [I (“我” in Chinese), mine (“我的” in Chinese), self (“自己” in Chinese), own (“自己的” in Chinese), and self (“本人” in Chinese)], and 5 words served as the *other* category [for male participants: he (“他” in Chinese), his (“他的” in Chinese), others (“别人” in Chinese), other’s (“别人的” in Chinese), and others (“外人” in Chinese); for female patients: she (“她” in Chinese), hers (“她的” in Chinese), others (“别人” in Chinese), other’s (“别人的” in Chinese), and others (“外人” in Chinese)]. Overall, 80 attribute (positive and negative) words were selected from the Chinese Affective Words System (16), matched in the number of Chinese characters, strokes, arousal, and familiarity. Through the combination of stimulus categories, the self-esteem GNAT consisted of four conditions (i.e., *self* + *positive*, *self* + *negative*, *other* + *positive*, and *other* + *negative*). The participants were asked to respond as quickly and accurately as possible to target stimuli (i.e., go task) by pressing the spacebar on the keyboard, or do nothing to the non-target stimuli (i.e., no-go task). Taking the *self* + *positive* condition as an example, the participants should press the spacebar when words of either *self* category or *positive* category are displayed on the screen but do nothing when words of either *other* category or *negative* category are displayed.

**FIGURE 1:**
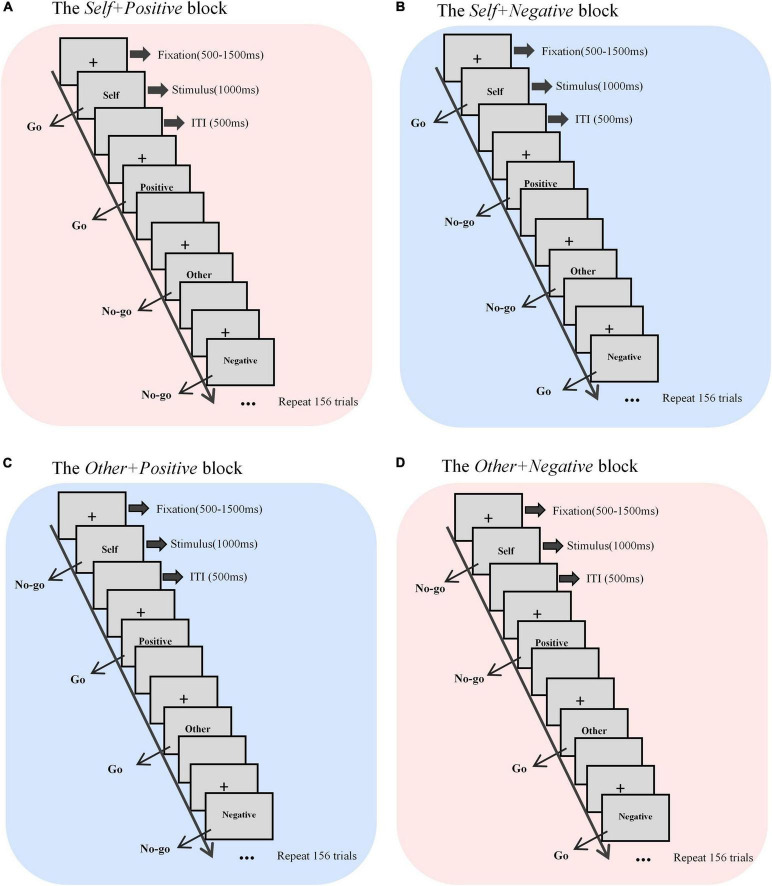
Sketch map of the self-esteem GNAT process. There were four blocks with different target stimuli, that is, **(A)** *self* + *positive*, **(B)** *self* + *negative*, **(C)** *other* + *positive*, and **(D)** *other* + *negative*. Each block contained 160 trials. ITI, inter-trial interval.

The self-esteem GNAT paradigm has a total of four blocks corresponding to the aforementioned four different conditions (Figure 1). At the beginning of each trial, there was a fixation (“+”) presented in the center of the screen, with a randomized duration between 500 and 1500 millisecond (ms). Then the word stimulus was displayed in the center of the screen, and the participants were required to respond to the go-task and do nothing to the no-Go task, as described before. Regardless of whether there was a response, the presentation time of each stimulus was fixed at 1000 ms, and the next trial began right after a 500-ms inter-trial interval (ITI). Each block had 160 trials, with each category (*self*, *other*, *positive*, and *negative*) of words displaying 40 times in a random order. Before each test block, the participants completed a practice block (10 trials) to ensure that they understood the rules. The participants were asked to complete all four blocks in a counterbalanced order between groups, with a 5-min break between each two blocks.

Behavioral indicators of interest mainly included the hit rate of the go-task under each condition and its corresponding response time (RT), which were calculated from the automatically recorded E-Data in E-Prime software. To exclude arbitrary answers, we also calculated the false alarm rate under the no-go task. If the participants arbitrarily presses the spacebar over and over to obtain a high hit rate in the go-task (e.g., *self-positive*), they would also get a high false alarm rate in the relative no-go task (e.g., *other negative*). To reflect participants’ ISE levels, D-scores for self-esteem were calculated using the method introduced in previous studies (25, 26). Briefly, we first calculated the difference of mean RTs between the *self-negative* condition and the *self-positive* condition and then divided the difference by the standard deviation (SD) for all RTs in these two conditions. Higher *self*-D-scores indicate stronger implicit bias toward *self-positive* association; that is to say, the higher the *self*-D-scores, the higher the level of ISE. Similarly, the D-score of “other esteem” was also calculated.

### EEG Recording and Analysis

To explore neural activity associated with ISE, EEG data were synchronously and continuously recorded during the self-esteem GNAT from a customized 64 Ag/AgCl channel EasyCap using a BrainAmp Standard recorder (Brain Products GmbH, Germany) at a 500-hertz (Hz) sampling rate. The FPz electrode was used as the recording reference and the left clavicle electrode as ground. The horizontal electrooculogram (HEOG) recording electrodes were placed 1 cm away from the outer corner of both eyes, and the vertical electrooculogram (VEOG) recording electrode was placed at the lower orbit of the left eye. Electrode impedances were kept below 5 kOhm (kΩ) during the recording.

Brain Vision Analyzer (version 2.0, Brain Products GmbH, Germany) was used for offline data analysis, according to established methods (26). In short, the EEG data were re-referenced to the averaged to the averaged left and right mastoids and band-pass-filtered between 0.1 and 30 Hz using a zero-phase shift Butterworth filter. A bad electrode was interpolated, and the independent component analysis (ICA) was applied to remove artifacts such as eye movement, myoelectricity, and electrocardiogram signals. For ERP analysis, continuous EEG data were segmented by a stimulus marker from −200 to 800 ms and then baseline-corrected using a −200 to 0−ms pre-stimulus. A given segment was rejected if the voltage gradient exceeded 50 microvolts (μV)/ms, the absolute amplitude was more than 75 μV, or the signal was flat (less than 0.5 μV for more than 100 ms). Finally, after a thorough manually check of artifacts, the individual ERPs for different categories of words in go tasks with correct responses were averaged separately.

Although the neural processes associated with ISE may involve more than a single ERP component, results from most of the previous research studies (13, 14, 27–29) in this field have suggested that the most critical one may be a late positive component (LPC) that begins approximately 300 ms after and continues to the end of the stimulation. Accordingly, we also focused on this component in the present study. In previous studies, researchers named this component P3 (or P300) or LPP. Task-relevant P3 is parietally maximal and often called P3b, whose amplitude is often considered to be related to task difficulty and effort devoted to the task and can be used as a measure of attention and other resource allocation. Many studies have drawn a conclusion that P3 amplitude is reduced in people with MDD (30, 31). LPP is a positive deflection that usually has the same onset time and scalp distribution as the P3 waveform (i.e., onset around 300 ms post-stimulation and the parietal maximum). LPP may become more centrally distributed over time, and its initial part may actually consist of an enlarged P3, reflecting an effect of the intrinsic task relevance of emotion-related stimuli (32). There is evidence that P3 and LPP are not identical (33); however, as a recent review concluded, they might reflect a common response to stimulus significance (34).

In the present study, by visual inspection of the grand averaged waveforms, we obtained such a positive potential that started about 300 ms and with a peak amplitude below 600 ms after the target stimuli. Evidence on whether P3 and LPP are the same ERP component remains controversial, whereas they may reflect output from a general system that tracks the time-course of stimulus importance (34). To this end, we named this P3/LPP waveform go-P3 in this study because our main interest was the neural correlates when participants made a correct response in go-tasks. Go-P3 was measured within a time window between 300 and 600 ms following the stimulus onset. ERP indicators of interest were mainly the peak amplitude of go-P3 that correctly responded under different conditions. Since the task-relevant P3 is parietally maximal and LPP may be more centrally distributed (32), we focused on centroparietal electrodes (Cz, CPz, and Pz) to better control the potential statistical error risk caused by multiple comparisons, referring to a recent study that employed P300 and LPP to investigate mechanisms of cognitive control training in MDD patients (35).

### Statistical Analyses

IBM SPSS Statistics version 22 (IBM Corp., Armonk, NY, United States) was used for data analysis. Comparisons of mean age, education, duration of illness, RSES scores, HAMD scores, and D-scores were conducted between the MDD group and the HC group with independent sample *t*-tests. D-scores of both groups were also independently compared to zero using the one-sample *t*-test. Comparisons of handedness and sex were conducted with the Pearson Chi-square test. A 2-group (MDD vs. HC) × 2-target (self vs. other) × 2-valence (positive vs. negative) mixed-model analysis of variance (ANOVA) was employed to compare the behavioral and ERP data, with group as a between-subject variable and target and valence as within-subject variables. The Greenhouse–Geisser method was employed to correct the degrees of freedom when the sphericity assumption was violated. Effect sizes were also estimated using partial eta-squared (η${}_{p}^{2}$). *Post hoc* analyses were conducted when a significant interaction was found, and Bonferroni correction was used to control possible type I error caused by multiple comparisons.

## Results

### Demographic and Clinical Characteristics

**FIGURE 2:**
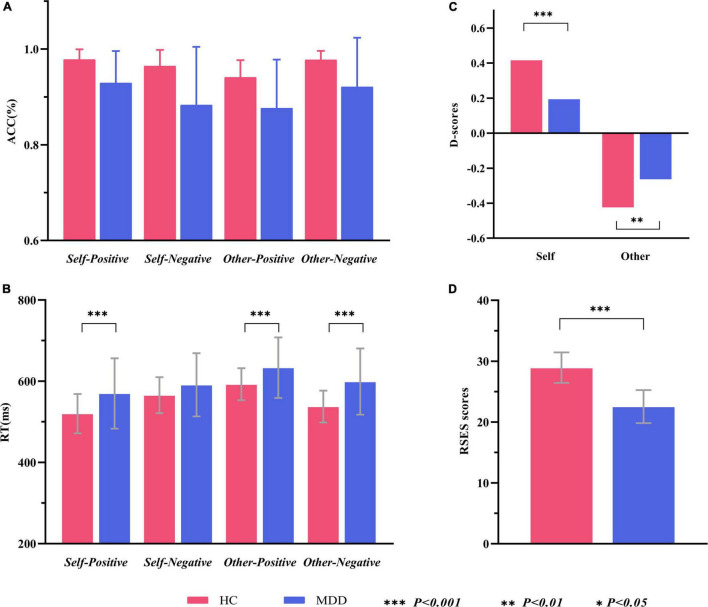
Within- and between-group comparisons of behavioral data in the self-esteem GNAT. **(A)** Grand average hit rate of go-task in different conditions and groups. **(B)** Grand average reaction time of go-task in different conditions and groups. **(C)** *Self-D* scores and *Other-D* socres in two groups. **(D)** Average RSES scores of two groups. ACC, accuracy (hit rate of go-task); RT, reaction time; RSES, Rosenberg Self-Esteem Scale.

In line with the inclusion and exclusion criteria, a total of 33 MDD patients and 32 HCs participated and finished this study. Data from one MDD patient and one HC were excluded because of technical reasons. Remaining data of 32 MDD patients (12 males and 20 females) and 31 HCs (15 males and 16 females) were analyzed. This sample size was sufficient to detect a medium-size effect with 80% power in mixed-model ANOVA. As shown in Table 1, there were no significant between-group differences in mean age, education level, handedness, and male-to-female ratio. The MDD group had a significantly lower RSES score than HCs (*t* = 9.733, *p* < 0.001), suggesting a decreased level of ESE in MDD (Figure 2D).

**TABLE 1:** Demographic characteristics and clinical information of two groups.

Variable	MDD (*n* = 32)	HC (*n* = 31)	Statistics	*p*-Value
Age range (years)	21–54	23–0	–	
Mean age (SD)	38.03 (9.12)	36.42 (6.80)	*t* = 0.784	0.431
Sex (M/F)	12/20	15/16	χ^2^ = 0.762	0.383
Handedness (R/M/L)	13/10/10	12/10/9	χ^2^ = 0.190	0.879
Year of education (SD)	11.69 (3.36)	12.74 (3.08)	*t* = 1.299	0.199
RSES (SD)	22.53 (2.71)	28.94 (2.50)	*t* = 90.733	<0.001
HAMD (SD)	29.47 (6.67)	–	–	
Duration of illness (year, SD)	3.66 (2.48)	–	–	

*MDD, major depressive disorder; HC, healthy control; SD, standard deviation; R, right; M, mixed; L, left; RSES, Rosenberg Self-Esteem Scale; HAMD, Hamilton Depression Scale.*

### Behavioral Data of Self-Esteem Go/No-Go Association Task

#### Accuracy and Response Times

The grand average hit rate of go-task and the false alarm rate of no-go tasks for both groups is shown in Table 2 and Figure 2A. For the hit rate of go-tasks, mixed-model ANOVA indicated no significant interaction effect for group × target × valence (*F*_1,61_ = 2.041, *p* = 0.158, η${}_{p}^{2}$ = 0.032), while a significant interaction between the target and valence (*F*_1,61_ = 24.722, *p* < 0.001, η${}_{p}^{2}$ = 0.288) was found. *Post hoc* analyses revealed that the participants had higher accuracy in the *self-positive* condition than in the *self-negative* condition (*p* = 0.007) but higher in the *other negative* condition than in the *other positive* condition (*p* < 0.001). The main effect of group was significant (*F*_1,61_ = 18.445, *p* < 0.001, η${}_{p}^{2}$ = 0.232), with higher accuracy for HCs than for MDD patients. For the false alarm rate of no-go tasks, a significant main effect for group (*F*_1,61_ = 11.418, *p* = 0.001, η${}_{p}^{2}$ = 0.158) was found, which showed that the HCs had a much lower false alarm rate than the MDD group. Overall, both groups had high hit rates in all go-tasks and low false alarm rates in the corresponding no-go tasks, indicating that no participants pressed the spacebar continuously (or never) during the tasks.

**TABLE 2:** Behavior data of two groups in the self-esteem GNAT.

Variable	Self + positive		Self + negative		Other + positive		Other + negative	
				
	HC	MDD	HC	MDD	HC	MDD	HC	MDD
Hit rate of go-task (SD)	0.98 (0.02)	0.93 (0.07)	0.96 (0.03)	0.88 (0.12)	0.94 (0.04)	0.88 (0.10)	0.98 (0.02)	0.92 (0.10)
False alarm rate of no-go task (SD)	0.04 (0.04)	0.08 (0.06)	0.03 (0.03)	0.05 (0.04)	0.07 (0.07)	0.13 (0.11)	0.02 (0.03)	0.04 (0.03)
RTs for hit (SD)	520.15 (48.50)	569.90 (86.50)	565.70 (44.42)	591.23 (77.96)	592.69 (39.51)	633.43 (74.62)	537.78 (39.10)	599.11 (81.50)

*HC, healthy control; MDD, major depressive disorder; RT, response time; SD, standard deviation.*

The average RTs for go tasks of two groups in different conditions are shown in Table 2 and Figure 2B. Mixed-model ANOVA indicated a significant interaction effect for group × target × valence (*F*_1,61_ = 10.545, *p* = 0.002, η${}_{p}^{2}$ = 0.147). *Post hoc* tests revealed significant higher RTs for the MDD group than for the HC group in the *self-positive* condition (*F*_1,61_ = 7.859, *p* = 0.007, η${}_{p}^{2}$ = 0.114), *other positive* condition (*F*_1,61_ = 7.265, *p* = 0.009, η${}_{p}^{2}$ = 0.106), and *other negative* condition (*F*_1,61_ = 14.351, *p* < 0.001, η${}_{p}^{2}$ = 0.190), but not in the *self-negative* condition (*F*_1,61_ = 2.528, *p* = 0.117, η${}_{p}^{2}$ = 0.040). The participants responded faster in the *self-positive* condition than in the *self-negative* condition (both *p* < 0.01), while slower in the *other positive* condition than in the *other negative* condition (both *p* < 0.01).

#### D-Scores

Figure 2C shows that comparisons between all D-scores and zero were significant (for MDD, *self* condition: *t* = 2.675, *p* = 0.012; *other* condition, *t* = 4.860, *p* < 0.001; for HCs, *self* condition, *t* = 9.805, *p* < 0.001; *other* condition, *t* = 8.837, *p* < 0.001). Interestingly, D-scores of the *self* condition (*self*-D-scores) were positive, while those of the *other* condition (*other* D-scores) were negative. Compared to the HCs, the MDD group had significantly lower *self*-D-scores (*t* = 3.516, *p* = 0.001), suggesting a remarkable decrease in ISE in MDD patients. The absolute *other* D-scores were larger in HCs than in MDD (*t* = 2.184, *p* = 0.033), indicating the implicit association between *other* and *negative* conditions in the MDD group is reduced. Because the direction of *self*-D-scores was opposite to that of *other* D-scores, we did not compare them from the perspective of target (self vs. other).

#### Event-Related Potential Data of Self-Esteem Go/No-Go Association Task

**FIGURE 3:**
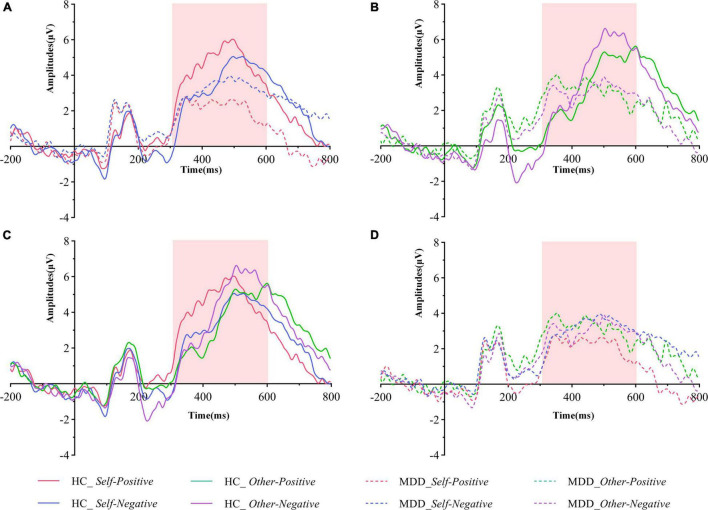
Grand averaged ERPs of both groups under different conditions of the pooling electrodes site (averaged Cz, CPz, and Pz sites). **(A)** Grand averaged ERPs in the HC group (solid lines) and the MDD group (dashed lines) during the self-positive (red lines) and the self-negative (blue lines) conditions. **(B)** Grand averaged ERPs in the HC group (solid lines) and the MDD group (dashed lines) during the other positive (green lines) and the self-negative (purple lines) conditions. **(C)** Grand averaged ERPs in the HC group during the self-positive (red lines), the self-negative (blue lines), the other positive (green lines), and the self-negative (purple lines) conditions. **(D)** Grand averaged ERPs in the MDD group during the self-positive (red lines), the self-negative (blue lines), the other positive (green lines), and the self-negative (purple lines) conditions. The go-P3 time windows are marked with light pink shadow. MDD, major depressive disorder; HC, healthy control.

**FIGURE 4:**
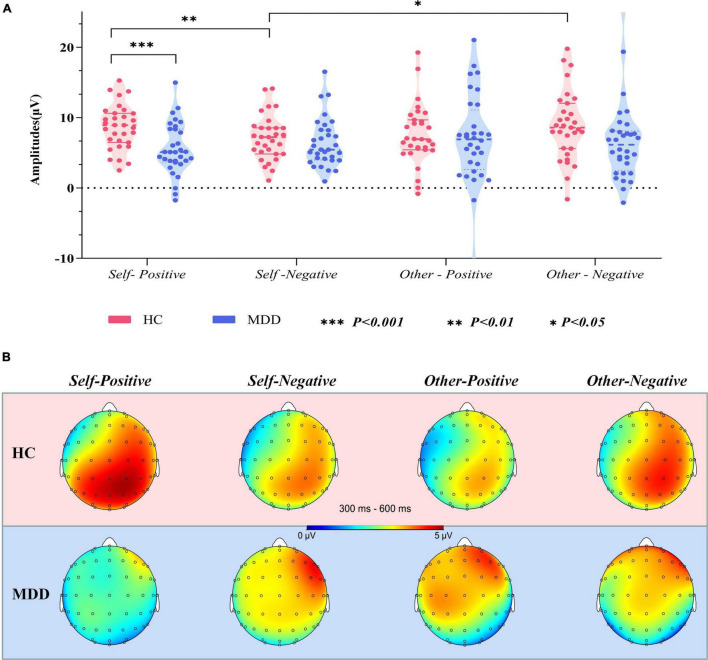
**(A)** Violin plots of pooled (Cz, CPz, and Pz) averaged go-P3 amplitudes in different groups and conditions. **(B)** Topographical distribution of grand averaged go-P3 within a time window of 300–600 ms post-stimuli under different conditions. MDD, major depressive disorder; HC, healthy control; SD, standard deviation.

The averaged peak go-P3 amplitude in centroparietal sites (Cz, CPz, and Pz) was analyzed by mixed-model ANOVA. The grand averaged amplitude of go-P3 that correctly responded to both groups under different conditions are shown in Figure 3. To better illustrate its overall distribution and within- and between-group differences, we also provided the topographical map and violin plots in Figure 4.

The interaction effect for group × target × valence was significant (*F*_1,61_ = 12.954, *p* = 0.001, η${}_{p}^{2}$ = 0.175). There was no significant interaction effect for group × target (*F*_1,61_ = 0.175, *p* = 0.677, η${}_{p}^{2}$ = 0.003), valence × group (*F*_1,61_ = 0.259, *p* = 0.612, η${}_{p}^{2}$ = 0.004), and target × valence (*F*_1,61_ = 1.858, *p* = 0.178, η${}_{p}^{2}$ = 0.030). The main effects of group (*F*_1,61_ = 3.582, *p* = 0.063, η${}_{p}^{2}$ = 0.055), target (*F*_1,61_ = 1.569, *p* = 0.215, η${}_{p}^{2}$ = 0.025), and valence (*F*_1,61_ = 0.034, *p* = 0.854, η${}_{p}^{2}$ = 0.001) were also not significant. *Post hoc* tests revealed that on the group level (MDD vs. HCs), the average amplitude in the *self-positive* condition was significantly smaller in MDD than in HCs (*F*_1,61_ = 13.401, *p* = 0.001, η${}_{p}^{2}$ = 0.180), while no such group difference was detected in the *self-negative* condition (*F*_1,61_ = 0.698, *p* = 0.407, η${}_{p}^{2}$ = 0.011). Moreover, in the HC group, go-P3 amplitude in the *self-positive* condition was larger than that in the *self-negative* condition (*F*_1,61_ = 7.655, *p* = 0.007, η${}_{p}^{2}$ = 0.111); remarkably, a reversed pattern was found in MDD groups where amplitude was smaller in the *self-positive* condition than in the *self-negative* condition, although this difference did not reach the significant threshold (*F*_1,61_ = 2.655, *p* = 0.108, η${}_{p}^{2}$ = 0.042). No significant difference was observed in both *other positive* and *other negative* conditions between groups (*p* = 0.671 and 0.076, respectively). On the target level (*self* vs. *other*), HCs demonstrated a larger go-P3 amplitude in the *other negative* condition than in the *self-negative* condition (*F*_1,61_ = 5.466, *p* = 0.023, η${}_{p}^{2}$ = 0.082), but no significant difference was found between the *self-positive* condition and the *other positive* condition (*F*_1,61_ = 2.228, *p* = 0.141, η${}_{p}^{2}$ = 0.035). For the MDD group, no significant difference was observed in aforementioned comparisons (both *p* > 0.05).

We also had a look at the go-P3 latencies; however, mixed-model ANOVA did not find significant interaction effect for group × target × valence. Furthermore, no significant second-order interaction effect or main effect was observed (all *p* > 0.05).

#### Correlations Among Indicators in Major Depressive Disorder

**FIGURE 5:**
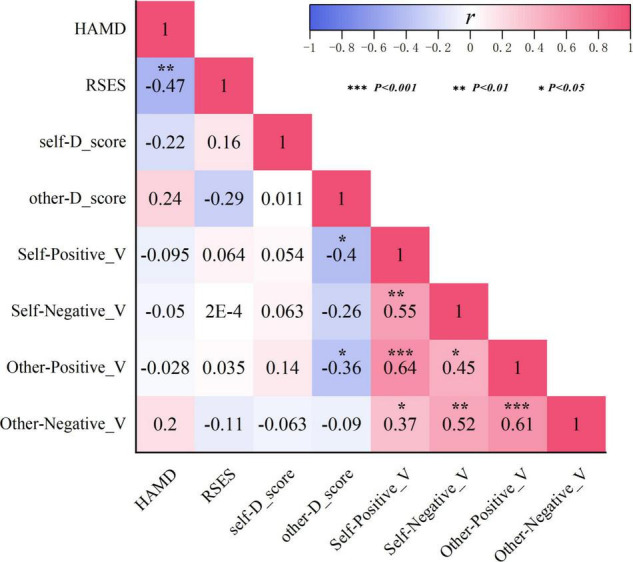
Correlations among indicators of HAMD scores, RSES scores, D-scores, and go-P3 amplitudes in the MDD group. HAMD, Hamilton Depression Rating Scale; RSES, Rosenberg Self-Esteem Scale.

The Pearson correlation analysis was employed to detect correlations among HAMD scores, RSES scores, D-scores, and go-P3 amplitudes (pooled by electrode sites) in the MDD group. Since there was no significant group difference in the go-P3 latencies, this indicator was not included here. As shown in Figure 5, HAMD scores were significantly negatively correlated with RSES scores (*r* = −0.47, *p* = 0.006), but not with either *self*-*r*-D-scores (*r* = −0.22, *p* > 0.05), *other* D-scores (*r* = 0.24, *p* > 0.05), or any of the go-P3 indicators (*r* = −0.095–0.2, all *p* > 0.05). In addition, RSES scores were also neither significantly correlated with *self*- and *other* D-scores (*r* = 0.16 and −0.29, respectively, both *p* > 0.05) nor with any of the go-P3 indicators (*r* = −0.15–0.22, all *p* > 0.05). *Other* D-scores were negatively correlated with the go-P3 amplitude under self-positive (*r* = −0.4, *p* = 0.002) and other positive conditions (*r* = −0.36, *p* = 0.004). In addition, there are broadly positive correlations among go-P3 amplitudes under different conditions (*r* = 0.37–0.64, all *p* < 0.05).

## Discussion

In the present study, in targeted clinically diagnosed patients with MDD, we found that both the ESE and ISE levels were reduced in this population. ISE indicators were not correlated with HAMD scores as ESE did, indicating that ISE may be more independent of clinical symptoms. Furthermore, we found that MDD patients, in comparison to HCs, had significantly smaller centroparietal go-P3 amplitude in the *self-positive* condition, but not in *self-negative* or both *other* conditions, suggesting that decreased neural processing that implicitly associate *self* with the *positive* condition might be important neural correlates of abnormal ISE in MDD.

It has been repeatedly reported that self-esteem is abnormal in depressive patients. Reduced ESE in MDD patients has been well established in previous studies; however, whether there exists lower ISE in this population remains unclear yet. Our results verified that the ESE level of MDD patients was reduced, as reflected by significant lower RSES scores in this group. As for ISE, we found a much lower level of *self*-D-scores in the MDD group. According to the calculation method mentioned previously, lower *self*-D-scores indicate higher implicit bias toward *self-negative* association, which represents a reduced level of ISE. This finding is consistent with those of previous studies. Discrepancies with some studies that failed to find a similar result might be due to differences in subject characteristics. For example, Risch et al. found that there was no significant difference in ISE between remitted depressive patients and HCs, but the times of depressive episodes would significantly aggravate the reduction of ISE (6). Smeijers et al. reported that remitted depressed patients demonstrated lower ESE, but not ISE, than never depressed controls (36). Different from these studies, the MDD patients in our study were all in their depressive episodes and had an average HAMD score closed to 30 points, corresponding to moderate level of severity. According to the dual process model, cognitive vulnerability to depression is observed when negatively biased associative (implicit, automatic, non-conscious, and intuitive) processing is uncorrected by reflective (explicit, controlled, conscious, and rational) processing (37, 38). In this way, it is understandable that lower ESE and lower ISE coexist in patients with major depressive episodes. Contrary to lower *self*-D-scores, we detected higher (less negative) *other* D-scores in MDD patients, indicating that when they view themselves negatively, they tend to have an increased bias to view others positively in relative terms. This result was in line with a previous large-sample size study and suggested that not only reduced ISE but also relatively increased implicit other esteem would play a role in depression (12).

We also found that MDD patients’ HAMD scores were significantly negatively correlated with their RSES scores, indicating that the more severe the depressive symptoms, the lower the ESE. This result was not surprising, since the RSES can be regarded as a comprehensive evaluation of some certain depressive symptoms, like “sense of inferiority,” which is common in MDD patients and is also assessed in the HAMD. Meanwhile, no significant correlation between the HAMD scores and *self*-D-scores was found, suggesting that ISE in MDD might be more independent of clinical symptoms. In this respect, abnormal ISE has more potential to be an endophenotype of MDD. As supporting evidence, a recent twin study (39) has proved the heritability of ISE, although the current mainstream view is that ISE is mainly determined by environmental factors. Our result further supported the assumption that ISE reflects different psychological constructs of self-esteem from ESE. In fact, previous studies have already shown that ISE and ESE having different neural bases. Using fMRI, Izuma et al. (40) found that although both ISE and ESE were related to neural signals in regions involved in self-processing, there were obvious differences; moreover, neural signals in reward-related brain regions were strongly related to ISE, but not to ESE. Together with previous evidence that ISE would have a better prediction value for relapse or recurrence of depression (10, 11), our data from psychological assessment and behavioral test, as well as their interconnections, provided new evidence that ISE may represent different aspects of self-esteem and have more potential to be an endophenotype of MDD. Further studies are also worth paying attention to the relations of ISE and other psychological characteristics relating to self-acceptance and negative clinical outcomes, such as affective temperaments (41).

We further investigated the neural correlates of abnormal ISE through the ERP technique, which can provide more direct information about brain activity during the GNAT than behavioral data. We found that the overall go-P3 amplitude of MDD was relatively smaller than that of HCs among different conditions (although in some cases, the between-group difference did not reach a significant threshold). In fact, it has been suggested that reduced P3 amplitude would play a central role in clinical depression, and reduced P3 amplitude in current depressive patients has been reported by numerous studies using different ERP paradigms that require quick response (42). Previous studies have revealed that many factors could influence the amplitude of the P3 component, mainly including the probability of target stimuli, the difficulty of task, the uncertainty of stimuli, and the amount of available resource allocation (43). In our GNAT paradigm, the frequency and probability among each task and condition are equal and counterbalanced. In addition, there were high correct response rates and low false alarm rate under different conditions in both groups. Therefore, it is unlikely that the overall reduction of P3 amplitude in the MDD group was caused by features of the GNAT paradigm itself. The reduction of P3 amplitude at the overall level has been largely interpreted as insufficient allocation of brain resources in this population (34, 42, 44), which is commensurate with the clinical phenomenon that people with depression often complain of slow thinking and reaction.

The most striking finding in the present study was that in the MDD group, the go-P3 amplitude under the *self-positive* condition was smaller than that under the *self-negative* condition, the pattern of which was just the opposite to that of HCs (Figure 3A). By contrast, the two groups had similar patterns under the *other* conditions (Figure 3B), that is, beyond a general decrease in neural reactivity, MDD patients were particularly less responsive to *self* items under the positive condition. Since P3 has been suggested as an index of ISE (13), the aforementioned findings would be important neural correlates of abnormal ISE in MDD. Our results coincided with those of previous studies. A recent study (15) using self-esteem IAT found that the self-positive condition induced smaller LPC amplitudes than the self-negative condition in dysphoric individuals, whereas the pattern was reversed in the control group. Another study also found that small LPC amplitudes existed in response to positive than to negative self-referent items in patients with current depression. Studies (16, 18) using the SRET paradigm also found that MDD patients had larger LPP to negative self-referent stimuli than to positive or neutral ones. Although these studies differ from the present study in terms of subjects’ clinical characteristics or experimental paradigm, their findings can be seen as supporting evidence for our results. In the present study, a larger go-P3 amplitude was observed in the *self-positive* condition relative to the *self-negative* condition for the HCs. As interpreted by previous studies, enhanced amplitude of P3/LPP was regarded as indicative of more voluntary attention and increased stimulus evaluation (28, 45). However, for the MDD group, go-P3 amplitude was smallest under the *self-positive* condition, suggesting that MDD patients were unable to engage similar voluntary attention and stimulus assessments under the *self-positive* condition. In other words, there was an implicit self-negativity bias in this population. Together with the lower *self*-D-scores in the MDD group (also indicates an implicit bias toward *self-negative* association), our finding provides support for Beck’s cognitive theory of depression (46). Previous studies have reported that ISE was robustly associated with reward-related brain regions (40). Moreover, patients with MDD tended to exhibit blunted amplitude of feedback-related negativity (FRN) in response to positive outcomes like monetary reward, rather than increased changes in response to negative outcomes (47). Our results have similarity to these findings, that is, changes in go-P3 amplitude are more obviously reflected as a decrease under *positive* conditions, rather than an increase under *negative* conditions. Thereby, fundamental processes of abnormal ISE in MDD patients may be mainly due to decreased attention and resource allocation toward positive stimuli.

Some limitations should be addressed in this study. First, we only focused on the go-P3 in this study, which might have missed some other valuable ERP components, such as no-Go N2, which is thought to be related to response inhibition. Moreover, the effect of medication on self-esteem in MDD participants may not be fully excluded, even if we have ruled out those who had recently taken any drugs known to affect the cognitive function. Similarly, the age range of the participants was broad, which might also have some potential influence on the results, although no significant group differences existed. In addition, due to the nature of the cross-sectional study and limited sample size, our results were unable to clarify the relationship between abnormal ISE and the process of MDD; therefore, well-designed longitudinal studies are warranted to address this question. Finally, neural processes underlying self-esteem are related to not only local cortical areas but also functional networks (48). Since ERPs have disadvantages in spatial resolution, other neuroimaging techniques with better balanced temporal and spatial resolution, such as functional near-infrared spectroscopy (fNIRS) or magnetoencephalography, are warranted to further exploring the neural mechanism of abnormal ISE in MDD.

## Conclusion

Taken together, by using a self-esteem GNAT paradigm and the ERP technique, our study verified that MDD patients had significantly lower ESE and ISE and found that the latter was more independent of clinical symptoms. Moreover, as indexed by the centroparietal go-P3 amplitude, we found that MDD patients exhibited decreased neural processing, which implicitly associates the *self* with the *positive* condition and relatively increased implicit association between the *self* and the *negative* condition. Our results provide new evidence for characteristics and underlying neural mechanisms of abnormal self-esteem in clinically diagnosed MDD patients, which also have some implications for optimizing treatment strategies, especially psychological intervention for this population.

## Data Availability Statement

The original contributions presented in the study are included in the article/supplementary material, further inquiries can be directed to the corresponding authors.

## Ethics Statement

The studies involving human participants were reviewed and approved by the Ethics Committee on Human Studies, The Affiliated Wuxi Mental Health Center of Nanjing Medical University. The patients/participants provided their written informed consent to participate in this study. Written informed consent was obtained from the individual(s) for the publication of any potentially identifiable images or data included in this article.

## Author Contributions

Z-HZ conceived the study and designed the study together with C-GJ and JW. C-GJ, HL, J-ZZ, and X-ZG recruited the subjects and collected the EEG and clinical data. C-GJ, Z-HZ, and JW performed the data analysis and drafted and revised the manuscript. All authors reviewed and commented on the final manuscript.

## Conflict of Interest

The authors declare that the research was conducted in the absence of any commercial or financial relationships that could be construed as a potential conflict of interest.

## Publisher’s Note

All claims expressed in this article are solely those of the authors and do not necessarily represent those of their affiliated organizations, or those of the publisher, the editors and the reviewers. Any product that may be evaluated in this article, or claim that may be made by its manufacturer, is not guaranteed or endorsed by the publisher.
